# Inequalities in Under-5 Mortality in Nigeria: Do Ethnicity and Socioeconomic Position Matter?

**DOI:** 10.2188/jea.JE20100049

**Published:** 2011-01-05

**Authors:** Diddy Antai

**Affiliations:** Division of Social Medicine, Department of Public Health, Karolinska Institute, Stockholm, Sweden; Division of Global Health & Inequalities, The Angels Trust, Stockholm, Sweden

**Keywords:** ethnicity, under-five mortality, soci-economic position, inequalities

## Abstract

**Background:**

Each ethnic group has its own cultural values and practices that widen inequalities in child health and survival among ethnic groups. This study seeks to examine the mediatory effects of ethnicity and socioeconomic position on under-5 mortality in Nigeria.

**Methods:**

Using multilevel logistic regression analysis of a nationally representative sample drawn from 7620 females age 15 to 49 years in the 2003 Nigeria Demographic and Health Survey, the risk of death in children younger than 5 years (under-5 deaths) was estimated using odds ratios with 95% confidence intervals for 6029 children nested within 2735 mothers who were in turn nested within 365 communities.

**Results:**

The prevalence of under-5 death was highest among children of Hausa/Fulani/Kanuri mothers and lowest among children of Yoruba mothers. The risk of under-5 death was significantly lower among children of mothers from the Igbo and other ethnic groups, as compared with children of Hausa/Fulani/Kanuri mothers, after adjustment for individual- and community-level factors. Much of the disparity in under-5 mortality with respect to maternal ethnicity was explained by differences in physician-provided community prenatal care.

**Conclusions:**

Ethnic differences in the risk of under-5 death were attributed to differences among ethnic groups in socioeconomic characteristics (maternal education and to differences in the maternal childbearing age and short birth-spacing practices. These findings emphasize the need for community-based initiatives aimed at increasing maternal education and maternal health care services within communities.

## INTRODUCTION

The association between ethnicity and health is well established.^1^^–^^3^ Recent reports have shown differences in mortality among ethnic groups in Nigeria.^4^^,^^5^ The existence of distinct cultural traits, values, beliefs, practices, and adaptive mechanisms to childhood morbidity and mortality among ethnic groups in Nigeria increases the need for better understanding of ethnic disparities in child survival.^6^ One possible explanation for ethnic differences in child survival in sub-Saharan Africa^7^ is that disparities in ethnic childhood mortality reflect differences in socioeconomic and demographic characteristics,^8^^–^^13^ which implies that ethnicity is confounded by socioeconomic position, and that ethnic childhood mortality differences result from cultural and traditional factors associated with ethnicity per se, suggesting that the norms, beliefs, and ideals of different ethnic groups are important regardless of socioeconomic status.^5^^,^^14^^,^^15^ In addition, ethnic group dominance of a nation’s political and economic institutions might enable certain ethnic groups to secure and maintain greater political power and advantage over other ethnic groups, thereby influencing child mortality. Exceptions to this include Nigeria and pre-war Rwanda, where economically disadvantaged groups held a monopoly on government leadership after independence.^16^^,^^17^

Nigeria is situated in West Africa and is bordered by Niger in the north, Chad in the northeast, Cameroon in the east, Benin in the west, and 850 kilometers of the Atlantic Ocean in the south. With a population of over 140 million people, Nigeria is the most populous country in Africa. It is geographically, culturally, and ethnically heterogeneous, with about 374 identifiable ethnic groups, of which the largest and politically dominant ethnic groups are the Hausa and Fulani of the north, the Yoruba of the southwest, and the Igbo of the southeast. These ethnic groups collectively account for 68% of the population. The Kanuri, Edo, Ijaw, Ibibio, Ebira, Nupe, and Tiv make up 27%, and other minority ethnic groups make up the rest.^16^ Cultural values and practices unique to these different ethnic groups influence child health outcomes.^18^^,^^19^ Yoruba and Igbo girls tend to marry in the third decade of life, while early marriage, before age 16 years, is common among the Hausa/Fulani/Kanuri ethnic groups.^19^ This commonly results in younger age at first birth, prolonged labor, and increased child mortality within these ethnic groups.^20^ The Hausa/Fulani/Kanuri ethnic groups also have a higher proportion of illiterate adults and less access to healthcare.^16^

This study investigated whether the effect of ethnicity on under-5 mortality is fully mediated by socioeconomic position. If there remain significant ethnic differences in under-5 mortality that are not explained by socioeconomic position, then socioeconomic position only partially mediates the association between ethnicity and under-5 mortality, and factors related to ethnicity can then be hypothesized as determinants. The objectives of this study were to assess whether under-5 mortality varies across socio-geographical contexts, to examine the individual-level relationship between ethnicity and under-5 mortality, and to determine whether contextual explanatory factors account for variation in under-5 mortality.

## METHODS

The data used in this study were from the 2003 Nigeria Demographic and Health Survey (DHS), which is a nationally representative survey that allows assessment of child health in a national context. The survey used a stratified 2-stage sampling procedure to select a probability sample of 7620 females according to a list of Standard Enumeration Areas from the 1991 Population Census sampling frame. A more detailed description is reported elsewhere.^21^ From these households, data were collected from 3725 females aged 15 to 49 years who had a total of 6029 live-born children within 5 years before the survey. Birth history data were used to compute under-5 mortality rates.

### Measures

#### Outcome

The outcome variable was the risk of under-5 death (0–59 months), defined as the probability of dying between birth and the fifth birthday.

#### Exposures

Ethnicity: Ethnicity of the mother was based on the answer to the question “What is your ethnic group?” in the DHS questionnaire, and was categorized as: (1) Hausa/Fulani/Kanuri, which were analyzed as group because these ethnic groups either speak a common language or dialect; share a common sense of identity, cohesion, or history; or have a single set of customs and behavioral rules, as in marriage, clothing, diet, taboos; (2) Igbo; (3) Yoruba; and (4) Others (ie, minor ethnic groups).

#### Individual-level risk factors

Seven individual-level variables of interest were examined:

(1) sex of the child
(2) birth order and interval between births, created by merging “birth order” and “preceding birth interval,” which were classified as: first births, birth order 2 to 4 after a short birth interval (<24 months), birth order 2 to 4 after a medium birth interval (24 to 47 months), birth order 2 to 4 after a long birth interval (≥48 months), birth order ≥5 after a short birth interval (<24 months), birth order ≥5 after a medium birth interval (24 to 47 months), and birth order ≥5 after a long birth interval (≥48 months). The variables birth order and preceding birth interval were merged because preceding birth interval, which is the interval between the previous birth and the birth of the child in question, excludes first births from the analysis since they are not preceded by another birth. Thus, the merger of these variables enables the analysis to include first births, which were merged with children born after a birth interval of 24 months or longer;
(3) maternal age, grouped as: 15 to 18, 19 to 23, 24 to 28, 29 to 33, and 34 years or older;
(4) maternal age at first birth, categorized as: 18 years or younger and 19 years or older;
(5) religion, categorized as: Christian, Muslim, and Traditional indigenous religion. Religion was included in order to examine the role of belief systems on ethnicity, as compared with other factors that might affect the influence of ethnicity on child survival;
(6) maternal education, categorized as: none, primary, and secondary or higher education; and
(7) maternal occupation, categorized as: professional/technical/managerial, clerical/sales/services/skilled manual, agricultural self-employed/agricultural employee/household or domestic/unskilled manual occupations, and not working.

#### Community-level risk factors

Community or contextual-level variables were analyzed at the level of the primary sampling unit (PSU), which are administratively defined areas used as proxies for neighborhoods or communities.^22^^,^^23^ PSUs are small and designed to be fairly homogeneous with respect to population socio-demographic characteristics, economic status, and living conditions. They comprise one or more enumeration areas, which are the smallest geographic units for which census data are available in Nigeria. Each cluster consists of a minimum of 50 households. If there were fewer than 50 households, a contiguous enumeration area was added.^21^ Two community-level variables were assessed here. The first was community maternal education (ie, the percentage of mothers with secondary or higher education in the PSU), which was categorized as low and high. This was assessed because maternal education is an important determinant of child survival in low- and middle-income countries^23^^,^^24^ and is associated with improved child survival and immunization rates.^25^^,^^26^ The second community-level variable was physician-provided community prenatal care, which was categorized as low and high based on the percentage of mothers who received prenatal care by a doctor. This was assessed because prenatal care directly increases mothers’ subsequent utilization of healthcare, such as institutional delivery and immunization.^27^^–^^30^

### Statistical analysis

The ethnic distribution of the children and mothers was estimated, and Pearson’s chi-square test was used to examine statistically significant differences, using Stata version 10.0 (StataCorporation).^31^

#### Multilevel logistic regression modeling

A 3-level multilevel logistic regression model was used to account for the hierarchical structure of the DHS data.^32^ Children (level 1) were nested within mothers (level 2) who were in turn nested within communities (level 3). Five models were fitted. Model 0 (null model) contained no explanatory variables and only focused on decomposing total variance into its individual and community components. Model 1 contained maternal ethnicity as the only variable. Model 2 added child-level variables (sex of the child and birth order/birth interval). Model 3 included mother-level variables (maternal age, maternal age at first birth, religion, maternal education, and maternal occupation). Models 4a and 4b included sequentially added community-level variables (community maternal education and community physician-provided prenatal care); one variable was added at a time in order to identify the community-level variable to which inequalities in under-5 mortality among the ethnic groups could be attributed.

The fixed effects (measures of association) were expressed as odds ratios (ORs) and 95% confidence intervals (95% CIs). The random effects (measures of variation) were expressed as variance partition coefficient (VPC) and percentage change in variance (PCV). The VPC is a measure of the extent to which siblings resemble each other more than they resemble children from other families in relation to under-5 mortality. A large VPC would indicate that mother and community factors are important in understanding the risk of under-5 mortality, while a VPC close to 0 would indicate that individual- and community-level factors exert only a small influence on under-5 mortality. Precision was evaluated by using the standard error (SE) of the explanatory variables. Parameters were tested in an approximate manner using the Wald statistic, ie, the ratio of the estimated variance to its standard error,^33^ and *P* values were calculated. MLwiN software 2.0.2 (Center for Multilevel Modeling)^34^ was used for the multilevel analyses, with binomial, predictive quasi-likelihood (PQL), and first-order linearization procedures, using Markov Chain Monte Carlo (MCMC) techniques.^35^ The Bayesian deviance information criterion (DIC) was used to estimate the goodness of fit of consecutive models; a lower DIC value indicates a better fit of the model.^36^

### Ethical considerations

The survey procedure and instruments for the 2003 Nigeria DHS were approved by the Ethics Committee of the Opinion Research Corporation (ORC) Macro International, Incorporated, Calverton, USA and by the National Ethics Committee of the Federal Ministry of Health, Nigeria. This study is based on analysis of secondary data; all participant identifiers were removed. Ethical permission for use of the data in the present study was obtained from ORC Macro Inc.

## RESULTS

### The demographic and socioeconomic characteristics of children and mothers in each ethnic group (Table 1)

The data were from 6029 children of 3725 mothers from 365 communities. The largest ethnic group was the Hausa/Fulani/Kanuri (43%), while the “other” composite ethnic group made up 35% of the sample. With respect to birth order/interval, maternal age, maternal education, and maternal age at first birth, the largest groups were children born in birth order 2 to 4 after an interval of 24 to 47 months (26%), age 24 to 28 years at child birth (30%), no maternal education (50%), and maternal age 18 years or younger at first birth (55%), respectively.

**Table 1.** Number and proportion of children in each ethnic group by demographic and socioeconomic characteristics

**Characteristics**	**Total** *n* (%)	**Ethnic affiliation**				

		**Hausa/Fulani/Kanuri** *n* (%)	**Igbo** *n* (%)	**Yoruba** *n* (%)	**Others** *n* (%)	***P*-value**
Ethnicity	6029 (100)	2589 (43)	732 (12)	577 (10)	2131 (35)	
Sex of the child						0.408
Male	3062 (51)	1293 (50)	385 (53)	306 (53)	1078 (51)	
Female	2967 (49)	1296 (50)	347 (47)	271 (47)	1053 (49)	
Birth order and birth interval in months						0.000
First birth (order 1)	1200 (20)	482 (19)	148 (20)	155 (27)	415 (20)	
2–4, <24	642 (11)	282 (11)	85 (12)	52 (9)	223 (11)	
2–4, 24–47	1563 (26)	629 (24)	182 (25)	178 (31)	574 (27)	
2–4, ≥48	416 (7)	119 (5)	60 (8)	76 (13)	161 (7)	
≥5, <24	484 (8)	268 (10)	54 (7)	6 (1)	156 (7)	
≥5, 24–47	1287 (21)	634 (24)	150 (21)	67 (12)	436 (20)	
≥5, ≥48	437 (7)	175 (7)	53 (7)	43 (7)	166 (8)	
Maternal age at birth, years						0.000
15–18	264 (4)	198 (8)	11 (1)	3 (0)	52 (2)	
19–23	1147 (19)	589 (23)	85 (12)	62 (11)	411 (19)	
24–28	1807 (30)	738 (28)	198 (27)	185 (32)	690 (33)	
29–33	1263 (21)	481 (18)	197 (27)	150 (26)	435 (20)	
34+	1548 (26)	587 (23)	241 (33)	177 (31)	543 (26)	
Maternal age at first birth, years						0.000
≤18	3337 (55)	1865 (72)	217 (30)	104 (18)	1151 (54)	
≥19	2692 (45)	724 (28)	515 (70)	473 (82)	980 (46)	
Religion						0.000
Christian	2307 (38)	12 (0.5)	673 (92)	298 (52)	1324 (62)	
Muslim	3598 (60)	2563 (99)	3 (0)	274 (47)	758 (36)	
Traditional indigenous	124 (2)	14 (0.5)	56 (8)	5 (1)	49 (2)	
Maternal education						0.000
None	3033 (50)	2020 (78)	125 (17)	85 (15)	803 (38)	
Primary	1473 (25)	349 (14)	255 (35)	174 (30)	695 (32)	
Secondary or higher	1523 (25)	220 (8)	352 (48)	318 (55)	633 (30)	
Maternal occupation						0.000
Not working	2074 (34)	1218 (47)	193 (26)	38 (7)	625 (29)	
Clerical, sales, services, skilled manual	2712 (45)	1227 (47)	292 (40)	405 (70)	788 (37)	
​ Agricultural self-employed or ​ employee, household & domestic, ​ unskilled manual	1013 (17)	99 (4)	212 (29)	82 (14)	620 (29)	
Professional/technical/management	230 (4)	45 (2)	35 (5)	52 (9)	98 (5)	

Among the Hausa/Fulani/Kanuri, children of birth order 2 to 4 born after an interval of 48 months or longer, and those of birth order 5 or higher born after an interval of 24 to 47 months were most common. The Hausa/Fulani/Kanuri mothers were most frequently 24 to 28 years of age, had no education, and were age 18 years or younger at the birth of their first child. Children of Igbo mothers were most commonly birth order 2 to 4 born after an interval of 24 to 47 months. The most common Igbo mothers were age 34 years or older at child birth, had a secondary or higher education, and were 19 years or older at first birth. Yoruba children were most commonly birth order 2 to 4 order born after an interval of 24 to 47 months, while Yoruba mothers were most commonly age 24 to 28 years at child birth, with a secondary or higher education, and were 19 years or older at first birth.

### Multilevel logistic regression analysis of individual- and community-level factors associated with under-5 mortality, by maternal ethnicity (Table 2)

#### Objective 1: To assess whether under-5 mortality varies across socio-geographical contexts

The null model (Model 0) indicated a significant variation in under-5 mortality across mothers (τ = 0.316, *P* = 0.021) and communities (τ = 0.253, *P* = 0.001), thereby justifying the use of multilevel models in this analysis. The intraclass correlations between mothers and communities, as indicated by the VPC, were 7% and 8.2% respectively, indicating that the variations in under-5 mortality were mostly due to child-level factors.

**Table 2.** Individual- and community-level contextual factors associated with under-5 mortality by maternal ethnicity in Nigeria from multivariable multilevel logistic regression models^±^

**Variables**	**Model 0** **(Empty model)**	**Model 1** **(Ethnicity)**	**Model 2** **(Child-level variables)**	**Model 3** **(Mother-level variables)**	**Model 4a** **(Community-level variables)**	**Model 4b** **(Community-level variables)**
	
	OR (95% CI)	OR (95% CI)	OR (95% CI)	OR (95% CI)	OR (95% CI)	OR (95% CI)
**Fixed effects**						
**Individual characteristics**						
Ethnicity^a^						
Igbo		0.60 (0.45–0.80)	0.62 (0.47–0.83)	0.64 (0.44–0.94)	0.66 (0.45–0.97)	0.78 (0.53–1.15)
Yoruba		0.43 (0.30–0.61)	0.49 (0.34–0.69)	0.88 (0.69–1.12)	0.89 (0.70–1.13)	0.82 (0.54–1.25)
Others		0.81 (0.68–0.98)	0.85 (0.71–1.02)	0.57 (0.39–0.84)	0.60 (0.40–0.89)	0.96 (0.75–1.23)
Sex of the child^b^						
Female			0.92 (0.79–1.07)	0.92 (0.79–1.07)	0.92 (0.79–1.07)	0.92 (0.79–1.07)
Birth order/birth interval in months^c^						
First birth (order 1)			1.36 (0.98–1.88)	1.54 (1.19–1.99)	1.55 (1.20–2.00)	1.59 (1.19–2.00)
2–4, <24			1.16 (0.87–1.53)	1.17 (0.88–1.56)	1.17 (0.88–1.55)	1.17 (0.88–1.55)
2–4, ≥48			0.76 (0.52–1.12)	0.71 (0.48–1.05)	0.71 (0.48–1.06)	0.74 (0.50–1.09)
≥5, <24			2.51 (1.92–3.27)	2.05 (1.51–2.80)	2.04 (1.49–2.78)	2.04 (1.50–2.78)
≥5, 24–47			1.23 (0.99–1.55)	1.00 (0.76–1.33)	1.00 (0.75–1.32)	1.00 (0.75–1.32)
≥5, ≥48			0.76 (0.53–1.10)	0.58 (0.38–0.88)	0.58 (0.38–0.88)	0.59 (0.37–0.89)
Maternal age at birth, years^d^						
15–18				0.75 (0.48–1.16)	0.74 (0.47–1.15)	0.73 (0.47–1.13)
19–23				0.98 (0.76–1.27)	0.98 (0.76–1.26)	0.96 (0.75–1.24)
29–33				1.02 (0.80–1.31)	1.03 (0.79–1.32)	1.02 (0.80–1.31)
≥34				1.31 (1.00–1.71)	1.32 (1.01–1.73)	1.33 (1.02–1.74)
Maternal age at first birth^e^						
≤18 years				0.98 (0.81–1.18)	0.98 (0.81–1.19)	0.98 (0.81–1.19)
Religion^f^						
Muslim				0.89 (0.68–1.16)	0.87 (0.67–1.14)	0.84 (0.64–1.10)
Traditional indigenous religion				1.70 (1.05–2.77)	1.67 (1.02–2.72)	1.57 (0.96–2.55)
Maternal education^g^						
None				2.01 (1.53–2.64)	1.87 (1.38–2.51)	1.80 (1.34–2.41)
Primary				1.76 (1.36–2.29)	1.66 (1.25–2.19)	1.63 (1.23–2.15)
Maternal occupation^h^						
Not working				1.00 (0.60–1.68)	1.00 (0.60–1.68)	1.01 (0.60–1.70)
Clerical, sales, services, ​ skilled manual				0.99 (0.59–1.64)	0.99 (0.59–1.64)	1.00 (0.60–1.67)
​ Agricultural self-employed or ​ employee, household & ​ domestic, unskilled manual				1.00 (0.58–1.72)	0.98 (0.51–1.69)	0.96 (0.56–1.66)
**Community characteristics**						
Community maternal education^i^						
Low					1.18 (0.90–1.53)	1.03 (0.79–1.34)
Physician-provided community prenatal care^j^						
Low						1.06 (0.85–1.31)
High						0.58 (0.44–0.77)
**Random effects**	**Empty**	**Ethnicity**	**Child**	**Mother**	**Community**	**Community**
Community-level						
Variance (SE)	0.253 (0.074) *P* = 0.001	0.202 (0.067) *P* = 0.003	0.196 (0.066) *P* = 0.010	0.160 (0.062) *P* = 0.010	0.137 (0.060) *P* = 0.009	0.137 (0.060) *P* = 0.022
VPC (%)	7	5.3	5.4	4.5	4.5	3.9
Explained variation (PCV) (%)	Reference	20.2	22.5	18.4	−1.2	15.4
Mother-level						
Variance (SE)	0.316 (0.137) *P* = 0.021	0.281 (0.132) *P* = 0.034	0.135 (0.126) *P* = 0.964	0.108 (0.123) *P* = 0.380	0.101 (0.122) *P* = 0.390	0.101 (0.122) *P* = 0.409
VPC (%)	8.2	7.4	3.7	3.0	3.0	2.9
Explained variation (PCV) (%)	Reference	11.1	57.3	20.0	1.8	−84.7
**Model fit statistics**						
DIC	4808	4792	4741	4714	4717	4702

Note: Model 0 contained no variables, Model 1 included ethnicity, Model 2 adjusted for birth order/birth interval, Model 3 additionally adjusted for maternal age at child birth and maternal education, Model 4a additionally adjusted for community maternal education, and Model 4b additionally adjusted for physician-provided community prenatal care.

Abbreviations: VPC, variance partition coefficient; DIC, deviance information criterion; SE, standard error; OR, odds ratio; CI, confidence interval.

^a^Hausa/Fulani/Kanuri; ^b^Male; ^c^Order 2–4, 24–47 months; ^d^24–28 years; ^e^19 years or more; ^f^Christian; ^g^Secondary or higher education; ^h^Professional/Technical/Management; ^i^High; ^j^Middle.

#### Objective 2: To elaborate the individual-level relationship between ethnicity and under-5 mortality

Ethnicity was included in Model 1 to investigate whether its effect was different across contexts. Crude ORs showed that, as compared with children of Hausa/Fulani/Kanuri mothers, children of Igbo mothers had a 40% lower risk of dying (OR = 0.60, 95% CI = 0.45–0.80), children of Yoruba mothers had a 57% lower risk of dying (0.43, 0.30–0.61), and children of mothers in other ethnic groups had a 19% lower risk of dying (0.81, 0.68–0.98). In comparison with the null model, the variation in under-5 mortality in Model 1 remained significant across mothers (τ = 0.281, *P* = 0.034) and communities (τ = 0.202, *P* = 0.003). The slightly increased intraclass correlation of 7.4 between mothers and the decreased intraclass correlation of 5.3 between communities indicate that controlling for ethnicity slightly increases the proportion of variance in under-5 mortality existing between mothers, but decreases the proportion of variance existing between communities. As indicated by the PCV, 11.1% and 20.2% of the variance in the odds of under-5 mortality across mothers and communities, respectively, was explained by ethnicity.

Adjusting for sex of the child and birth order/birth interval in Model 2 attenuated the risks of dying for children of Igbo (OR = 0.62, 95% CI = 0.47–0.83) and Yoruba mothers (0.49, 0.34–0.69), as compared with the Hausa/Fulani/Kanuri ethnic group. In comparison with Model 1, the mother-level variation became nonsignificant, while the community-level variation decreased further, but remained significant (τ = 0.196, *P* = 0.003). The intraclass correlation between mothers decreased to 3.7%, while the intraclass correlation between communities remained basically unchanged at 5.4%. In this model, 57.3% and 22.5% of the variance in the odds of under-5 mortality across mothers and communities, respectively, was explained by birth order/birth interval.

Maternal age, maternal age at first birth, maternal religious affiliation, maternal education, and maternal occupation were introduced in Model 3. The risk for children of Igbo mothers remained basically unchanged, the risk for children of Yoruba mothers became nonsignificant, and the risk for children of mothers from other ethnic groups was attenuated (OR = 0.57, 95% CI = 0.39–0.84). In comparison with Model 2, the community-level variation decreased further while remaining significant (τ = 0.160, *P* = 0.010). The intraclass correlation between mothers decreased slightly to 4.5%, while the intraclass correlation between communities decreased to 3.0%. The decreased variance in the odds of under-5 mortality of 3.0% and 4.5% across mothers and communities, respectively, was explained by mother-level characteristics.

#### Objective 3: Determine whether contextual explanatory variables account for ethnic variation in under-5 mortality

Finally, community maternal education and physician-provided community prenatal care were added in Models 4a and 4b, respectively. With the introduction of community maternal education (Model 4a), the point estimates remained basically the same. However, with the introduction of physician-provided community prenatal care (Model 4b), the differences in the risks of under-5 mortality between the ethnic groups became statistically nonsignificant, indicating that the association between ethnicity and under-5 mortality could be attributed to differential access to prenatal care provided by doctors within communities. In addition, model 4b showed that the risk of dying was 59% higher for first births (OR = 1.59, 95% CI = 1.19–2.00) and 2-fold for children of birth order ≥5 after a short birth interval (<24 months) (OR = 2.04, 95% CI = 1.50–2.78), but 41% lower for children of birth order ≥5 after a long birth interval (≥48 months) (OR = 0.59, 95% CI = 0.37–0.89). The risks were 33% higher for children of mothers who were age 34 years or older (1.33, 1.02–1.74), as compared with children of mothers age 24 to 28 years. The risks of dying were 63% and 80% higher for children of mothers with primary (1.63, 1.23–2.15) or no education (1.80, 1.34–2.41), respectively, as compared with children of mothers with secondary or higher education.

In comparison with Model 3, community-level variation in Models 4a and 4b decreased further while remaining significant (τ = 0.137, *P* = 0.022). The intraclass correlation between communities remained at 4.5% in Model 4a and 3.9% in Model 4b, while the intraclass correlation between mothers was 3.0 in Model 4a and 2.9 in Model 4b, in comparison with the previous models. This means that differences in the under-5 mortality between ethnic groups are partly due to community-level characteristics in Models 4a and 4b. Community-level and mother-level factors explained 15.4% and −84.7% of the variance in the odds of under-5 mortality, respectively. The decrease in DIC value with each successive model indicates a good fit between the model and the data.

## DISCUSSION

### Summary of findings

With respect to the objectives of the study, the findings were as follows:

(1) Under-5 mortality was highest among children of Hausa/Fulani/Kanuri mothers and lowest among children of Yoruba mothers.
(2) Under-5 mortality varied significantly across contexts, thereby justifying the use of multilevel modeling in the analysis.
(3) There was significant variation in under-5 mortality by ethnicity, much of which was explained by differences in individual-level explanatory factors (birth order/birth interval and socioeconomic characteristics, ie, maternal age and maternal education) and community-level explanatory factors (physician-provided community prenatal care).

The findings of this study indicate that much of the association between maternal ethnicity and under-5 mortality was mediated by differences in individual-level socioeconomic characteristics, especially maternal education and maternal age. Ethnicity is thus an explanatory factor for differences in health-seeking behavior, since it was significantly associated with maternal education even after controlling for socioeconomic factors. Similar findings have been reported in other studies in Nigeria^37^ and elsewhere.^38^^,^^39^ The proportions of individual-level characteristics in this study showed that Hausa/Fulani/Kanuri mothers were most commonly uneducated, younger, and age 18 years or younger at first birth; this supports the association found in this study between socioeconomic position and under-5 mortality.

The results also showed that ethnic differences in under-5 mortality were associated in part with birth order/birth interval, which in turn supports hypotheses suggesting that ethnic differences in health-seeking behavior explain health disparities.^40^^,^^41^ This finding reflects the influence of cultural beliefs and practices in childbirth fertility-related behaviors most commonly associated with the Hausa/Fulani/Kanuri ethnic group of Northern Nigeria. The culture of early marriage, young age at first birth, high order births with short birth intervals, and low literacy and educational attainment among females of the Hausa/Fulani/Kanuri ethnic group significantly contributes to under-5 mortality differences among the ethnic groups, a point that has also been stressed in a recent study in Nigeria.^42^ Furthermore, maternal education is also known to influence the chance that mothers will access health care services, such as physician-provided prenatal care, hospital delivery, and child immunization. Thus, ethnicity may have an indirect effect on under-5 mortality by influencing physician-provided community prenatal care, as was the case in this study and other studies.^40^^,^^41^

As in previous studies,^24^^,^^25^^,^^43^ first births and higher order births were significantly associated with higher risks of under-5 deaths. As discussed earlier, this is more commonly associated with health behaviors of individuals from the Hausa/Fulani/Kanuri ethnic group, and is also linked with the socioeconomic level of the mothers. It was expected that higher maternal age (34 years or older) would be associated with a lower risk of under-5 mortality, as this association had been reported.^26^ It is believed that older mothers are more educated, have better jobs, and may be better empowered to partake in household decision-making and fertility-related decisions. Lower maternal education (primary education or less) was significantly associated with an increased risk of under-5 death, which is in line with results from other studies.^27^^,^^37^ The reason for this is that higher education enables mothers to acquire modern and improved perceptions of disease causes and treatment, improves the health and welfare of their child, and enhances decision-making power.

Physician-provided community prenatal care was significantly associated with under-5 mortality in this study: the risk of dying was lower for children of mothers residing in communities with high levels of physician-provided prenatal care, as compared with children of mothers residing in communities with low levels of such care. Physician-provided community prenatal care is also an indication of the access and quality of care received by the mother and infant during delivery. Moreover, it reflects how socioeconomic position at the community level affects health outcomes and parallels the associations seen at the individual level. The presence of significant community-level variation after controlling for individual- and community-level characteristics indicates a need for further exploration of community-level effects on under-5 mortality risks.

### Policy implications

This study showed that ethnic differences in the risks of under-5 deaths were attributable to differences in socioeconomic characteristics among the ethnic groups and to differences in the health-seeking behavior of the different ethnic groups. This reflects the need for interventions to reduce economic inequalities among women, both at individual and community levels. There is a need for community-based initiatives aimed at increasing the proportion of mothers with higher education in communities and the proportion of mothers who receive maternal health care services (prenatal care and subsequently hospital delivery) in communities. Furthermore, attempts must be made to change health-damaging cultural norms and behaviors that are more common among certain ethnic groups, such as short birth-spacing intervals (especially among younger mothers). Interventions aimed at eliminating ethnic disparities in child health in Nigeria should be incorporated into public health objectives and program planning at the national level.

This study has some limitations. First, the administratively defined boundaries used as proxies for neighborhoods may nondifferentially misclassify individuals into inappropriate administrative boundaries, which can generate information biases and reduce the validity of the analyses. Second, other individual- and community-level factors not addressed in the present study, such as cultural beliefs, may also be important determinants of full immunization; these were excluded because of the absence of relevant data.
